# Expression, tumor immune infiltration, and prognostic impact of HMGs in gastric cancer

**DOI:** 10.3389/fonc.2022.1056917

**Published:** 2022-12-07

**Authors:** Zhiheng Wu, Yang Huang, Weiwei Yuan, Xiong Wu, Hui Shi, Ming Lu, Aman Xu

**Affiliations:** ^1^ Department of General Surgery, the First Affiliated Hospital of Anhui Medical University, Hefei, Anhui, China; ^2^ Department of General Surgery, Anhui Public Health Clinical Center, Hefei, Anhui, China; ^3^ School of Optometry and Ophthalmology and the Eye Hospital, Wenzhou Medical University, PR China, State Key Laboratory of Optometry, Ophthalmology, and Visual Science, Wenzhou, Zhejiang, China; ^4^ Department of Immunology, School of Basic Medical Sciences, Anhui Medical University, Hefei, Anhui, China

**Keywords:** Expression, HMGs, gastric cancer, Prognostic biomarkers, TCGA

## Abstract

**Background:**

In the past decade, considerable research efforts on gastric cancer (GC) have been expended, however, little advancement has been made owing to the lack of effective biomarkers and treatment options. Herein, we aimed to examine the levels of expression, mutations, and clinical relevance of HMGs in GC to provide sufficient scientific evidence for clinical decision-making and risk management.

**Methods:**

GC samples were obtained from The Cancer Genome Atlas (TCGA). University of California Santa Cruz (UCSC) XENA, Human Protein Atlas (HPA), Gene Expression Profiling Interactive Analysis (GEPIA), Kaplan-Meier Plotter, cBioPortal, GeneMANIA, STRING, LinkedOmics, and DAVID databases were employed. The “ggplot2” package in the R software (×64 3.6.3) was used to thoroughly analyze the effects of HMGs. qRT-PCR was performed to assess HMG levels in GC cell lines.

**Results:**

A total of 375 GC tissues and 32 paraneoplastic tissues were analyzed. The levels of HMGA1, HMGA2, HMGB1, HMGB2, HMGB3, HMGN1, HMGN2, and HMGN4 expression were increased in GC tissues relative to normal gastric tissues. HMGA1, HMGA2, HMGB1, HMGB2, and HMGB3 were highly expressed in GC cell lines. The OS was significantly different in the group showing low expressions of HMGA1, HMGA2, HMGB1, HMGB2, HMGB3, HMGN2, HMGN3, and HMGN5. There was a significant difference in RFS between the groups with low HMGA2, HMGB3, and high HMGN2 expression. The levels of HMGA2, HMGB3, and HMGN1 had a higher accuracy for prediction to distinguish GC from normal tissues (AUC value > 0.9). HMGs were tightly associated with immune infiltration and tumor immune escape and antitumor immunity most likely participates in HMG-mediated oncogenesis in GC. GO and KEGG enrichment analyses showed that HMGs played a vital role in the cell cycle pathway.

**Conclusions:**

Our results strongly suggest a vital role of HMGs in GC. HMGA2 and HMGB3 could be potential markers for prognostic prediction and treatment targets for GC by interrupting the cell cycle pathway. Our findings might provide renewed perspectives for the selection of prognostic biomarkers among HMGs in GC and may contribute to the determination of the optimal strategy for the treatment of these patients.

## Introduction

Gastric cancer (GC) is a common and lethal malignancy of the digestive system. GC is highly aggressive and poses a major health burden worldwide (1). According to the latest data published by GLOBOCAN in 2020, 10,89,103 new cases of GC were diagnosed globally, and it is estimated that 768,793 people suffer from GC, accounting for 7.7% of the overall cancer mortality rate (2). Although the diagnosis and therapeutic strategies for GC are continuously optimized, the current treatment for GC is focused on surgery combined with chemotherapy and radiotherapy (3). Although considerable research efforts in GC have been made in the last few decades, little advancement has been made because of the lack of effective biomarkers and treatment options. Therefore, identifying new specific molecular biomarkers for the diagnosis and prognosis of GC patients is necessitated, which may help facilitate the development of targeted diagnostic and therapeutic strategies (4).

High-mobility groups (HMGs) of proteins occur widely in eukaryotic cells. These are the most abundant group of chromatin proteins, second only to eukaryotic histones, and are critical to eukaryotic gene regulation (5). These play an essential role in the chromatin structure and function as well as the regulation of gene expression (6, 7). Based on the molecular mass, sequence similarity, and DNA structural properties, HMGs can be further divided into three subfamilies (8, 9)— HMGA, HMGB, and HMGN. The HMGA subfamily includes HMGA1 and HMGA2; HMGB subfamily includes HMGB1, HMGB2, and HMGB3, and the HMGN subfamily includes HMGN1, HMGN2, HMGN3, HMGN4, and HMGN5.

Since the discovery of HMGA1 in human cervical cancer (HeLa) cells in 1983 (10), increasing evidence suggests that the HMGA1 protein is a master modulator and plays a critical role in the normal development and tumor progression of various malignancies (11–13). The AT-hook DNA-binding domain defines the HMGA family, comprising HMGA1 and HMGA2 proteins, and these mediate the binding to AT-rich regions of chromatin (14, 15). Upon binding to DNA, the DNA structure is altered and the assembly of transcriptional complexes or “enhanced progeny” is coordinated to regulate gene expression (16, 17). Previous studies have shown that HMGA1 and HMAG2 are involved in regulating several cellular processes, including gene transcription, cell cycle progression, embryonic development, tumor transformation, differentiation, aging, viral integration, and DNA repair, owing to their interaction with other proteins, binding to the DNA, and regulation of gene expression (5, 18–21). Previous findings suggest that both HMGA1 and HMGA2 serve as useful biomarkers and therapeutic targets for malignancy (22, 23).

The high mobility group protein B (HMGB) family (including HMGB1, HMGB2, and HMGB3) regulates DNA replication, transcription, recombination, and repair mechanisms and functions as cellular factors mediating responses to infection, injury, and inflammation (24, 25). The high-mobility group protein B1 (HMGB1) was originally thought of as a ubiquitous nuclear protein involved in the maintenance of nucleosome integrity and promoting gene transcription. However, since then, HMGB1 has been reevaluated as a quintessential damage-associated molecular pattern (DAMP) protein along with its exogenous counterpart, the pathogen-associated molecular pattern (PAMP), the alarm system of the body, functions to prevent disruption of homeostasis (26). HMGB1 is an oncogene in GC, and GC patients with high HMGB1 expression have a poor prognosis (27, 28). Knocking down HMGB1 inhibits the growth and invasion of GC cells through the NF-κB pathway both *in vitro* and *in vivo (* 29). HMGB2 down-regulates the NF-κB axis to reduce inflammatory damage (30), and miR-329 inhibits melanoma progression by down-regulating HMGB2 *via* the β-catenin pathway (31). HMGB2 is a confirmed downstream target of miR-23b-3p (32) and lncRNA MALAT1 (33). HMGB3 is localized in the nucleus, cytoplasm, and chromosome and primarily expressed in embryonic and bone marrow hematopoietic stem cells, whereas in the normal adult tissues, its expression is low to negligible (34). Aberrant expression of HMGB3 is closely associated with the development of many tumors; HMGB3 is highly expressed in various cancers [including breast cancer (35), gastric cancer (36), nasopharyngeal carcinoma (37), and esophageal cancer (38)] and is associated with the incidence of advanced tumors and a low survival rate of these patients. HMGB3 is not only involved in tumorigenesis, malignant proliferation, metastasis, and cell cycle regulation but also in regulating the development of chemoresistance (39, 40). Taken together, previous findings suggest that HMGB1, HMGB2, and HMGB3 are potential tumor diagnostic and prognostic marker proteins (41).

The high mobility group nucleosome binding protein (HMGN) family is a class of non-histone chromatin-building proteins localized in the nuclei of almost all mammals and most vertebrates. These can change the structure of chromatin, enhance the transcription and replication of chromatin templates, and participate in cellular activities like DNA replication and expression, cell differentiation, organ development, and gene expression regulation (42–45). The HMGN family comprises five proteins— HMGN1, 2, 3, 4, and 5 (44). HMGN1 and HMGN2 affect DNA damage repair and organ development and maturation by regulating the expression of genes or proteins (46, 47), and are also implicated in tumor immune responses (48, 49). HMGN3 is closely related to the occurrence of type 2 diabetes in humans and chemotherapeutic resistance in liver cancer (to vinblastine, topotecan, paclitaxel, adriamycin, etc.); thus, HMGN3 is a preventive target for type 2 diabetes (50) and can enhance the efficacy of chemotherapy in patients with liver cancer (51). HMGN4 and HMGN2 are similar, and both code an intronless gene (52). HMGN4 is relatively poorly studied and has only been reported in thyroid tumors (53) and breast cancer (54). HMGN5 not only binds to nucleosomes to regulate the chromosomal structure and function, thus affecting DNA replication and repair but also plays a role in tumor development, with its overexpression being crucial in tumor cell invasion and metastasis (55). The regulation of autophagy HMGN5 is key in the development of chemoresistance and provides a novel target for improving osteosarcoma therapy (56). Therefore, the HMGN family is a potential tumor diagnostic and prognostic marker protein and an emerging novel target for the clinical treatment of tumors.

To date, no study has systematically evaluated the function of the HMGs in GC using bioinformatic methods. Herein, we aimed to examine the level of expression, mutational profile, and clinical significance of HMGs in GC, thereby providing reliable scientific evidence for clinical decision-making and risk management.

## Materials and methods

### Gene expression difference analysis

Genes expression difference analysis was performed using the R software (×64 3.6.3). The Cancer Genome Atlas (TCGA) and Genotype-Tissue Expression Project (GTEx) databases were utilized. Pan-Cancer TPM data [TOIL workflow processed (57)] were obtained from the University of California Santa Cruz (UCSC) Xena Browser (https://xenabrowser.net/datapages/). RNA-seq data of TCGA-STSD in FPKM format were used. The RNA-seq data in the FPKM format was converted to the TPM format and log2 transformation was performed. Differential expressions between GC and normal tissues were visualized using the “ggplot2” package in R, using an independent sample t-test for group comparisons. P values < 0.05 were considered statistically significant.

### Cell culture

Normal gastric epithelial cells (GES-1) and GC (AGS, HGC-27, MGC-803, BGC-823, MKN-45, and MKN-28) cell lines were obtained from the Cell Bank of the Chinese Academy of Sciences (Shanghai, China). All GC cell lines were grown in RPMI1640 (Wisent Corporation, Nanjing, China) medium containing 10% fetal bovine serum FBS (Wisent Corporation, Nanjing, China), 100U/ml penicillin, and 100μg/ml streptomycin (Beyotime, Shanghai, China) at 37℃ in a humidified atmosphere containing 5% CO_2_.

### Quantitative real-time polymerase chain reaction (qRT-PCR) analysis

Following the manufacturer’s guide, total RNA was extracted from GC cells using an RNA-Quick Purification Kit (RN001, Esunbio, Shanghai, China). The extracted RNA was used to reverse transcribed to the corresponding cDNA using Hifair^®^ III 1st Strand cDNA Synthesis SuperMix for qPCR (gDNA digester plus) (Yeasen, Shanghai, China), and qRT-PCR analysis was performed using the Hieff^®^ qPCR SYBR Green Master Mix (High Rox Plus)(Yeasen, Shanghai, China). Thermal cycling conditions were as follows: denaturation at 95°C for 5 min, followed by 40 cycles of denaturation at 95°C for 10 sec, and extension at 60°C for 30 sec. Glyceraldehyde-3-phosphate dehydrogenase (GAPDH) was used as an internal control and the results were normalized to its expression. Fold changes in mRNA expression were calculated using the comparative Ct method (ΔΔCt). Primer pairs for target genes used in the qRT-PCR assay are listed in **Table 1**.

**Table 1:** Sequences of primer pairs for target genes used in the qRT-PCR.

Gene		Sequence (5' to 3')
HMGA1	Forward	GAAGTGCCAACACCTAAGAGACC
	Reverse	GGTTTCCTTCCTGGAGTTGTGG
HMGA2	Forward	GAAGCCACTGGAGAAAAACGGC
	Reverse	GGCAGACTCTTGTGAGGATGTC
HMGB1	Forward	GCGAAGAAACTGGGAGAGATGTG
	Reverse	GCATCAGGCTTTCCTTTAGCTCG
HMGB2	Forward	GGTGAAATGTGGTCTGAGCAGTC
	Reverse	CCTGCTTCACTTTTGCCCTTGG
HMGB3	Forward	CCAAGAAGTGCTCTGAGAGGTG
	Reverse	CTTCTTGCCTCCCTTAGCTGGT
HMGN1	Forward	ACCTCCTGCAAAAGTGGAAGCG
	Reverse	GTTTCTTGGTTAGCCACTTCGGC
HMGN2	Forward	AAACCTGCTCCTCCAAAGCCAG
	Reverse	CTTGCCAGCATCAGCTTTTCCC
HMGN3	Forward	CACAAGACGGTCTGCCAGATTG
	Reverse	CCTCCTTCTTCCCTTTAGCACC
HMGN4	Forward	CACAGAGGAGATCAGCTCGGTT
	Reverse	GGTTGTTCCCATCCTTTCCAGC
HMGN5	Forward	CTTGTGCCAGTTACACCAGAGG
	Reverse	TCAGCAACTGCTTGGGCACTTG
GAPDH	Forward	GTCTCCTCTGACTTCAACAGCG
	Reverse	ACCACCCTGTTGCTGTAGCCAA

### The human protein atlas (HPA)

The images of immunohistochemistry staining for GC and normal tissues were obtained from HPA (https://www.proteinatlas.org/). HPA provides a diverse protein landscape by integrating various histological techniques, including mass spectrometry-based proteomics, transcriptomics, and systems biology, to map all human proteins in cells, tissues, and organs. For protein expression analysis, in HPA, sections from cancer tissue microarrays were immunohistochemically stained and corresponding slides were scanned to generate digital images. These were analyzed.

### Gene expression profiling interactive analysis (GEPIA)

GEPIA 2 (http://gepia2.cancer-pku.cn/#index) is a multi-dimensional cancer genomic dataset comprising a large amount of data from TCGA and GTEx databases. GEPIA 2 (Expression DIY platform) was adopted to evaluate the association between HMGs and the clinical stage, and Pearson’s correlation coefficient was employed to assess statistical results.

### Kaplan-Meier plotter

The Kaplan-Meier plotter assesses the impact of several genes on the prognosis of different cancer types (http://kmplot.com). The GC samples were categorized into two groups according to the levels of HMG expression. Overall survival (OS) was defined as the time to death or the last follow-up after the initial diagnosis of GC, whichever occurred earlier. Recurrence-free survival (RFS) was the time to recurrence after diagnosis. The hazard ratio (HR) and P-values were determined. P values < 0.05 were considered statistically significant.

### The receiver operating characteristic (ROC) curves

The ROC curve is a comprehensive index reflecting the continuous variables of sensitivity and specificity, and their interrelationship is suggested by conformation analysis. The ROC curve analysis was performed for the RNA-seq data in TCGA-STSD FPKM format (FPKM format was converted to TPM format and log2 transformation was performed) using the R software (×64 3.6.3). The ROC curves for markers of imaging for OS were constructed, and the areas under the ROC curves (AUC) were evaluated empirically using the trapezoid rule utilizing the “pROC” and “ggplot2” R packages. The pROC package (version 1.17.0.1) was used for analysis and the ggplot2 package (version 3.3.3) was used for visualization.

### cBioPortal

cBioPortal was employed to perform gene variation analysis in GC (http://www.cbioportal.org/), including amplification, mutation, and copy number variants. An overview of the genetic alterations in each HMGs was provided to visualize the complete details of each mutation type per sample.

### GeneMANIA

GeneMANIA (http://www.genemania.org) is a database that generates hypotheses on gene functions, analyzes gene lists, and prioritizes genes for functional testing based on functions with high accuracy according to prediction algorithms. We used it to weigh the predictive value of the indicated HMGs.

### STRING

STRING (https://string-db.org/) provides information on protein interactions, including direct physical interactions among proteins and indirect functional correlations between proteins. The purpose was to achieve a thorough and objective worldwide network and propose a unique set of computational projections. Protein-protein interaction (PPI) network analysis was performed using STRING to collect and integrate the manifestations and potential interactors of HMGs.

### Immune infiltration analysis

The recognition and infiltration of immune cells in tumors play an essential function in cancer detection and elimination. The “GSVA” package in R software (×64 3.6.3) (58) and the ssGSEA algorithm, along with RNA-seq data in TCGA-STSD FPKM format (FPKM format was converted to TPM format and log2 transformation was performed) were used for immune infiltration analysis and visualization of the results. The immune cells (59) included activated dendritic cells (aDCs), B cells, CD8 T cells, cytotoxic cells, DCs, eosinophils, immature dendritic cells (iDCs), macrophages, mast cells, neutrophils, NK CD56bright cells, NK CD56dim cells, NK cells, plasmacytoid dendritic cells (pDCs), T cells, T helper, Tcm (T central memory), Tem (T effector memory), Tfh (T follicular helper), Tgd (T gamma delta), Th1 cells, Th17 cells, Th2 cells, and Treg cells. The correlation between HMGs and immune cells was assessed by Spearman’s correlation analysis. The subgroup comparisons of HMGs and immune infiltrates were statistically analyzed using Weltch’s t-test.

### Heat map correlational analyses

The association of each of the two HMGs and the most relevant genes among the HMGs as well as the association between HMGs and immune checkpoints were estimated by Spearman’s correlation coefficient. Statistical analysis was performed using the R software (×64 3.6.3) and plots were drawn. A heat map was drawn to show the top 50 genes that were significantly relevant to HMGs. The immune checkpoints included PDCD1 [ENSG00000188389]; CD274 [ENSG00000120217]; PDCD1LG2 [ENSG00000197646]; CTLA4 [ENSG00000163599]; LMTK3 [ENSG00000142235]; LAG3 [ENSG00000089692]; TIGIT [ENSG00000181847]; HAVCR2 [ENSG00000135077], and SIGLEC15 [ENSG00000197046]. A P-value under 0.05 was regarded as a significant correlation.

### Gene ontology (GO) and Kyoto encyclopedia of genes and genomes (KEGG) analyses

GO and KEGG enrichment analyses were performed using the DAVID database (https://david.ncifcrf.gov/). GO terms include three classifications, namely biological processes (BPs), cellular components (CCs), and molecular functions (MFs). The DAVID database provides calculations for significantly enriched pathways. The plots for GO and KEGG analyses were graphed using the R package, ggplot2 (×64 3.6.3).

## Results

### Levels of mRNA expression of HMGs in human cancers

**Figure 1:**
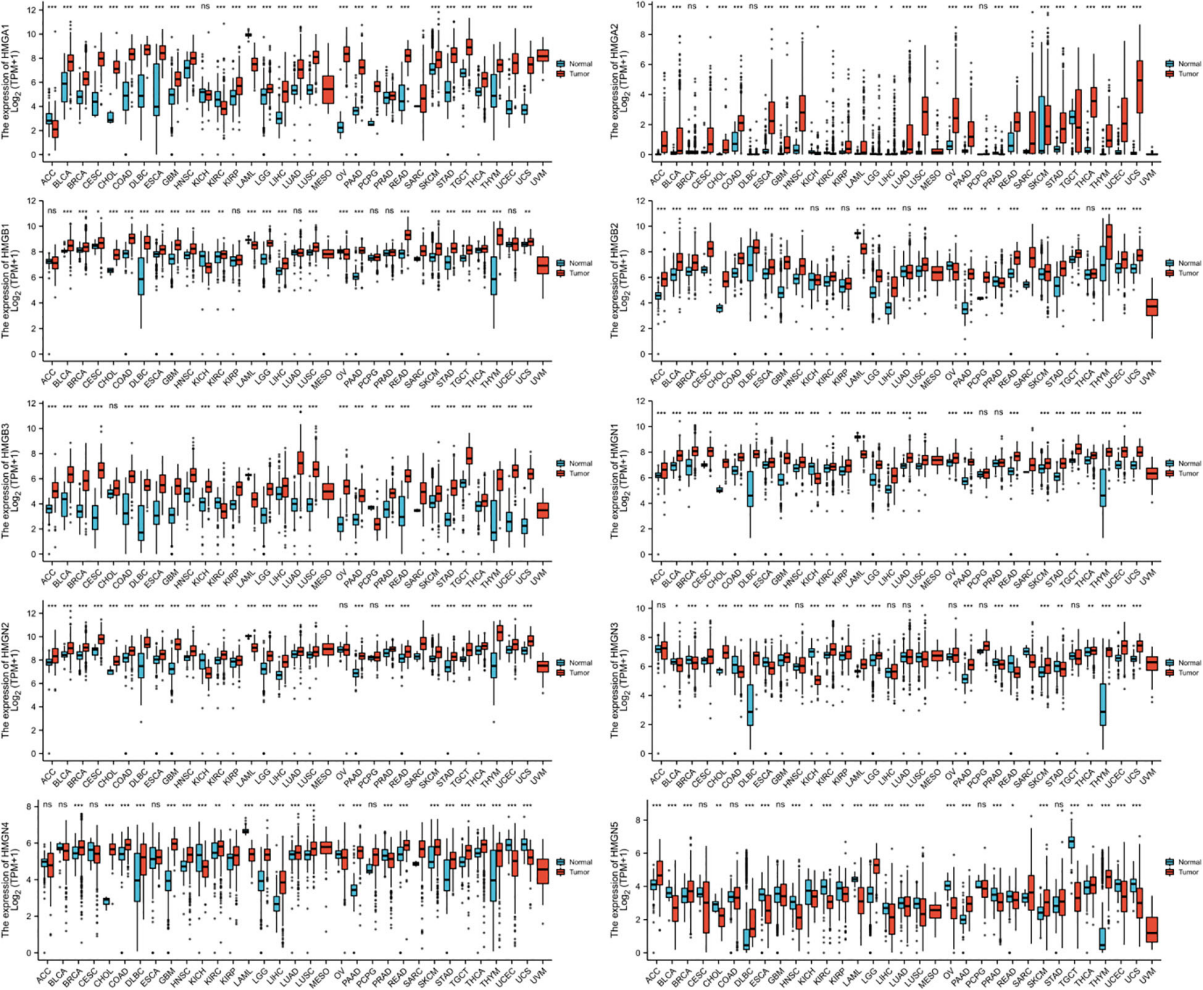
mRNA expression levels of HMGs in different human cancer types. The numbers of Normal group (N) and Tumor group (T) in different types of cancer were: ACC (Adrenocortical Carcinoma): N: 128, T: 77; BLCA (Bladder Urothelial Carcinoma): N: 28, T: 407; BRCA (Breast Invasive Carcinoma): N: 292, T: 1099; CESC (Cervical Squamous Cell Carcinoma and Endocervical Adenocarcinoma): N: 13, T: 306; CHOL (Cholangio Carcinoma): N: 9, T: 36; COAD (Colon Adenocarcinoma): N: 349, T: 290; DLBC (Lymphoid Neoplasm Diffuse Large B-cell Lymphoma): N: 444, T: 47; ESCA (Esophageal Carcinoma): N: 666, T: 182; GBM (Glioblastoma Multiforme): N: 1157, T: 166; HNSC (Head and Neck Squamous Cell Carcinoma): N: 44, T: 520; KICH (Kidney Chromophobe): N: 53, T: 66; KIRC (Kidney Renal Clear Cell Carcinoma): N: 100, T: 531; KIRP (Kidney Renal Papillary Cell Carcinoma): N: 60, T: 289; LAML (Acute Myeloid Leukemia): N: 70, T: 173; LGG (Brain Lower Grade Glioma): N: 1152, T: 523; LIHC (Liver Hepatocellular Carcinoma): N: 160, T: 371; LUAD (Lung Adenocarcinoma): N: 347, T: 515; LUSC (Lung Squamous Cell Carcinoma): N: 338, T: 498; MESO (Mesothelioma): N: 0, T: 87; OV (Ovarian Serous Cystadenocarcinoma): N: 88, T: 427; PAAD (Pancreatic Adenocarcinoma): N: 171, T: 179; PCPG (Pheochromocytoma and Paraganglioma): N: 3, T: 182; PRAD (Prostate Adenocarcinoma): N: 152, T: 496; READ (Rectum Adenocarcinoma): N: 318, T: 93; SARC (Sarcoma): N: 2, T: 262; SKCM (Skin Cutaneous Melanoma): N: 818, T: 469; STAD (Stomach Adenocarcinoma): N: 210, T: 414; TGCT (Testicular Germ Cell Tumors): N: 165, T: 154; THCA (Thyroid Carcinoma): N: 338, T: 512; THYM (Thymoma): N: 446, T: 119; UCEC (Uterine Corpus Endometrial Carcinoma): N: 101, T:181; UCS (Uterine Carcinosarcoma): N: 78, T: 57; UVM (Uveal Melanoma): N: 0, T: 79. (*p< 0.05; **p<0.01; ***p<0.001; NS, no statistically significant difference).

The levels of mRNA expression of each subfamily of the HMG family in cancer and para-cancerous tissues were characterized utilizing TCGA database (**Figure 1**). The results showed that compared to para-cancerous tissues, the mRNA expression of all members of the HMG family was higher in most cancers except for HMGN5 which showed a consistently low expression across most cancer tissues.

### Levels of mRNA and protein expression of HMGs in GC

**Figure 2:**
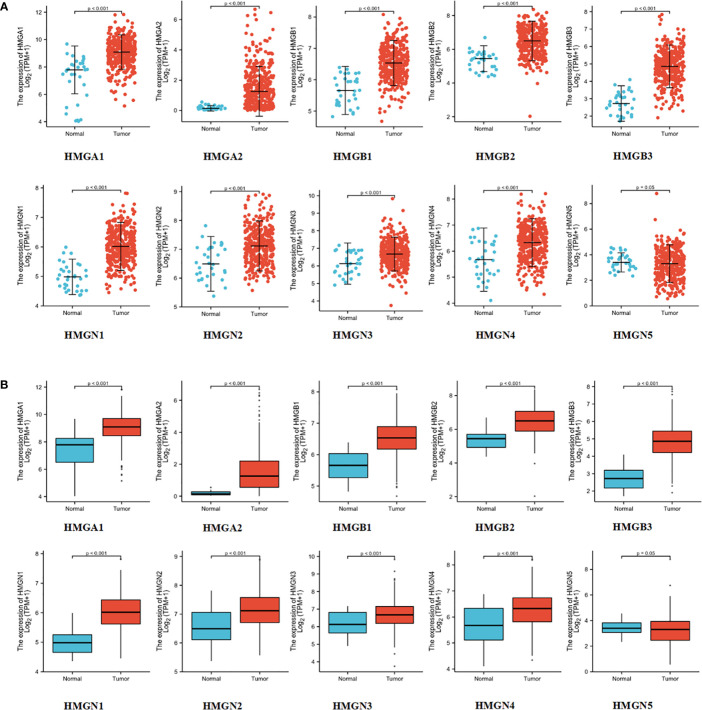
mRNA expression levels of HMGs in GC: **(A)** Scatter diagram; **(B)** Box plot.

To test the levels of mRNA expression of HMGs in GC, we analyzed the transcriptomic data from TCGA database (**Figures 2A, B**) comprising 375 GC tissues and 32 para-cancerous tissues. The expression of HMGN5 decreased slightly compared to the para-cancerous tissues, while that of other HMGs increased significantly (p<0.05).

**Figure 3:**
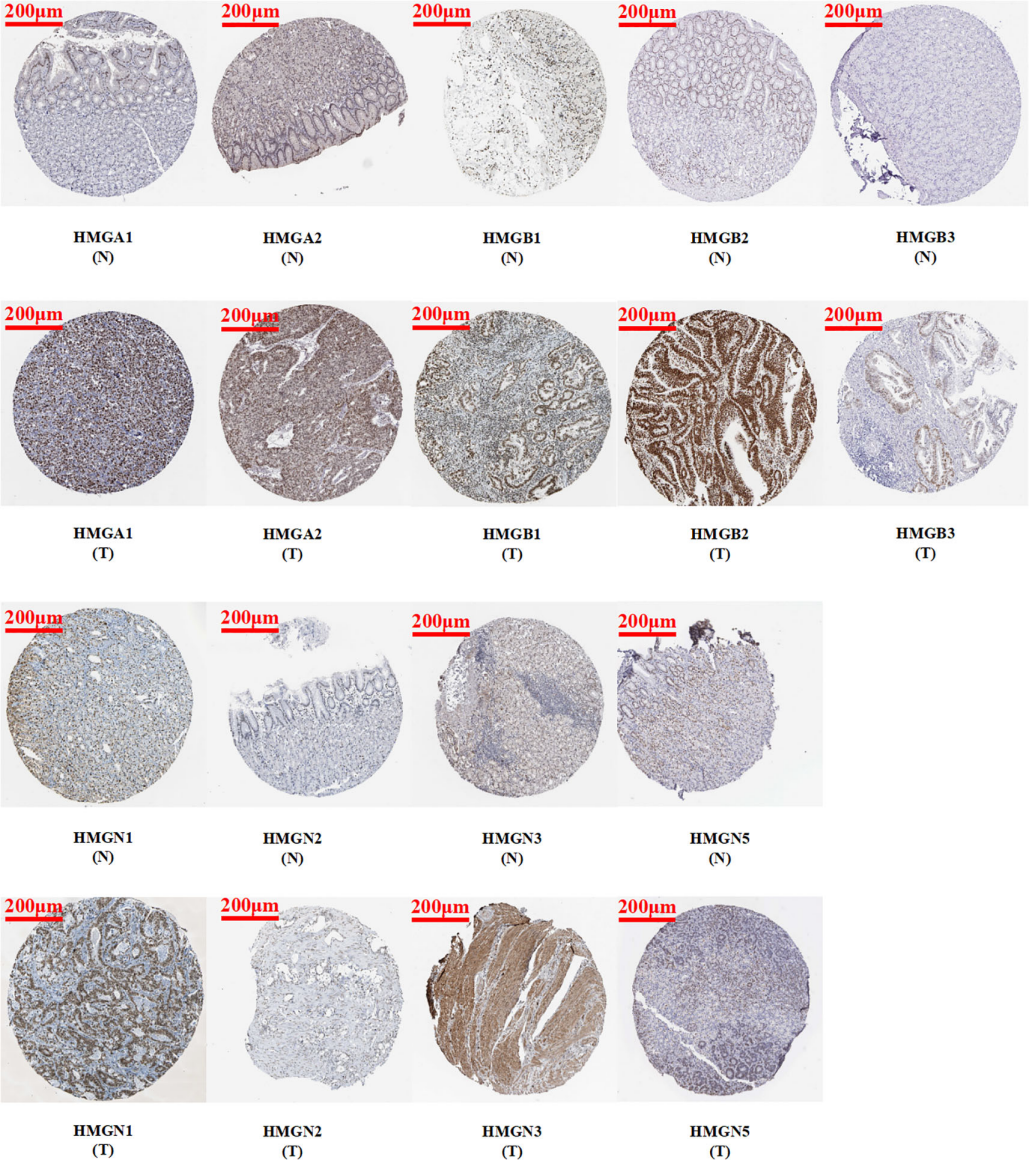
Protein expression levels of HMGs in GC. Images of immunohistochemistry staining for GC and normal tissues were collected with HPA. The greater the antigen content (representing the level of protein expression) and the higher the distribution density, the stronger the positive result color rendering. According to the degree of color rendering of positive markers, they are classified as: blue, negative; light yellow, weakly positive; brown, moderately positive; and dark brown, strongly positive. (scale bar = 200 μm).

Immunohistochemistry is based on the principle of specific binding of antigens and antibodies which aids the visualization of expression and localization of a protein. It intuitively reveals the expression of the protein in the tissue. The protein levels of HMGs in GC (images of immunohistochemistry staining for GC and normal tissues were collected from HPA) were investigated (**Figure 3**). Except for the absence of information on HMGN4, the protein levels of HMGA1, HMGA2, HMGB1, HMGB2, HMGB3, HMGN1, HMGN2, and HMGN3 were elevated, while that of HMGN5 was similar in GC tissues compared to para-cancerous tissues.

### Relative expression of HMGs in GC cell lines

**Figure 4:**
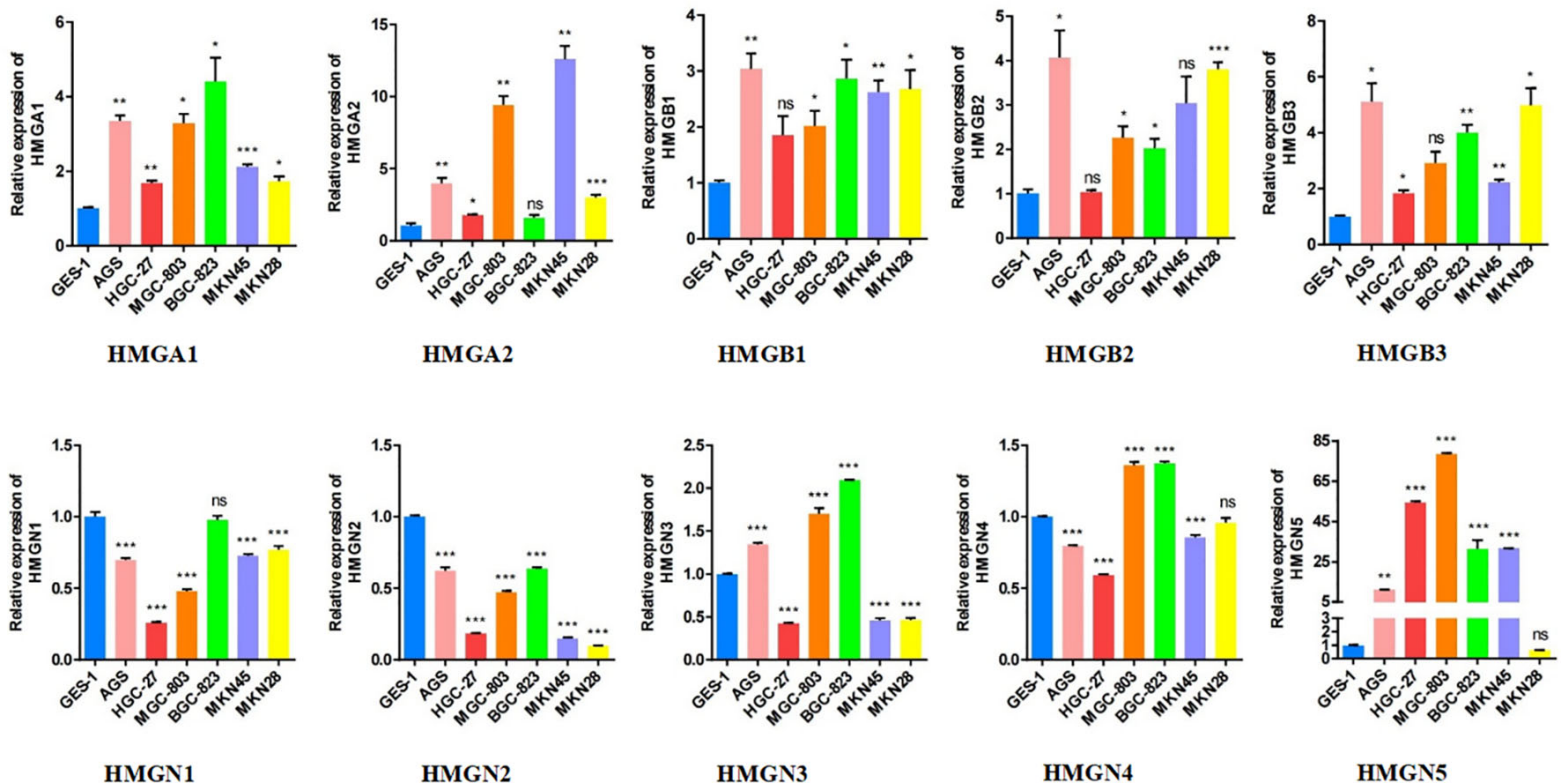
The relative expression levels of HMGs in GC cell lines (AGS, HGC-27, MGC-803, BGC-823, MKN-45, MKN-28) were detected by qRT-PCR (*p< 0.05; **p<0.01; ***p<0.001; NS, no statistically significant difference).

Subsequently, we performed qRT-PCR analysis to verify the expression of HMGs across GC cell lines (**Figure 4**). The results demonstrated that the expressions of HMGA1, HMGA2, HMGB1, HMGB2, and HMGB3 were high in GC cell lines (AGS, HGC-27, MGC-803, BGC-823, MKN-45, and MKN-28) compared to the normal gastric epithelial cells (GES-1), consistent with previous predictions using TCGA cohort. However, the mRNA levels of both HMGN1 and HMGN2 were low in the GC cell lines, contrary to our prediction. Given that the protein levels of HMGN1 and HMGN2 were elevated, their translation from mRNA to protein may be subjected to some post-transcriptional regulation that correspondingly up-regulated their expression. HMGN3 was highly expressed in AGS, MGC-803, and BGC-823 cells but its levels were low in the remaining cells. The expression profile of HMGN4 was similar to that of HMGN3, with elevated expression in MGC-803 and BGC-823 cells and low expression in the remaining cell lines, inconsistent to an extent with our predictions. This suggested the existence of regulatory processes during protein translation. Interestingly, except for MKN28, HMGN5 was highly expressed across GC cell lines, which was exactly the opposite of our prediction, indicating the necessary occurrence of protein translational modifications (PTMs) in HMGN5 mRNA, resulting in correspondingly lower expression levels of its protein.

### Association between clinical characteristics and HMGs

**Figure 5:**
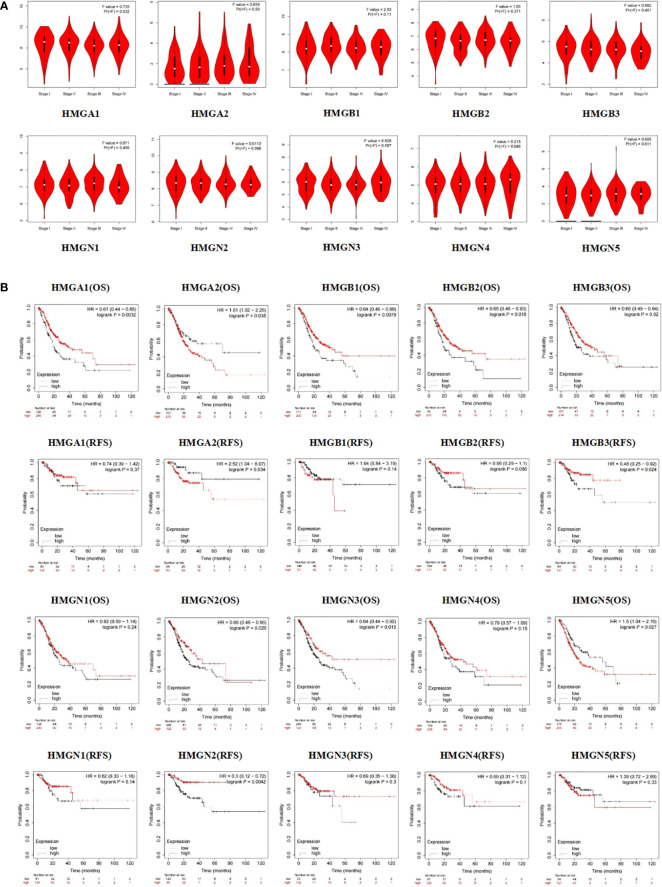
**(A)** Association between HMGs and tumor stage in GC; **(B)** Effect of HMGs on OS and RFS in GC.

Subsequently, we evaluated the association between differentially expressed HMGs and the pathological stage of GC patients. The relationship between tumor stage and HMGs was examined using the GEPIA database (**Figure 5A**). No significant correlations were observed between HMGs and tumor stage, suggesting that HMGs might not be associated with the pathological stages of GC.

Apart from the tumor stage, Kaplan-Meier Plotter was employed to assess the impact of HMGs on GC prognosis. As shown in **Figure 5B**, the OS of the high HMGA1 expression group was higher than that of the low expression group (p < 0.05), while HMGA1 expression was not significantly correlated with RFS. HMGB1 and HMGN3 showed the same trends for GC prognosis as HMGA1. The HMGA2 low-expression group showed remarkably better OS and RFS. The OS of the HMGB2 high expression group was higher than that of the low expression group (p < 0.05) but the difference in RFS between the two groups was statistically insignificant, which might be due to the small sample size. Similar to HMGA1, both OS and RFS were higher in the high HMGB3 and HMGN2 expression groups than in the corresponding low expression groups (p < 0.05). In contrast, the high HMGN5 expression group had worse OS compared to the low expression group (p < 0.05) while HMGN5 expression was not significantly correlated with RFS. The expressions of HMGN1 and HMGN4 showed no significant effects on the prognoses of GC patients (both OS and RFS).

### Correlation between HMGs and potential diagnostic markers for GC

**Figure 6:**
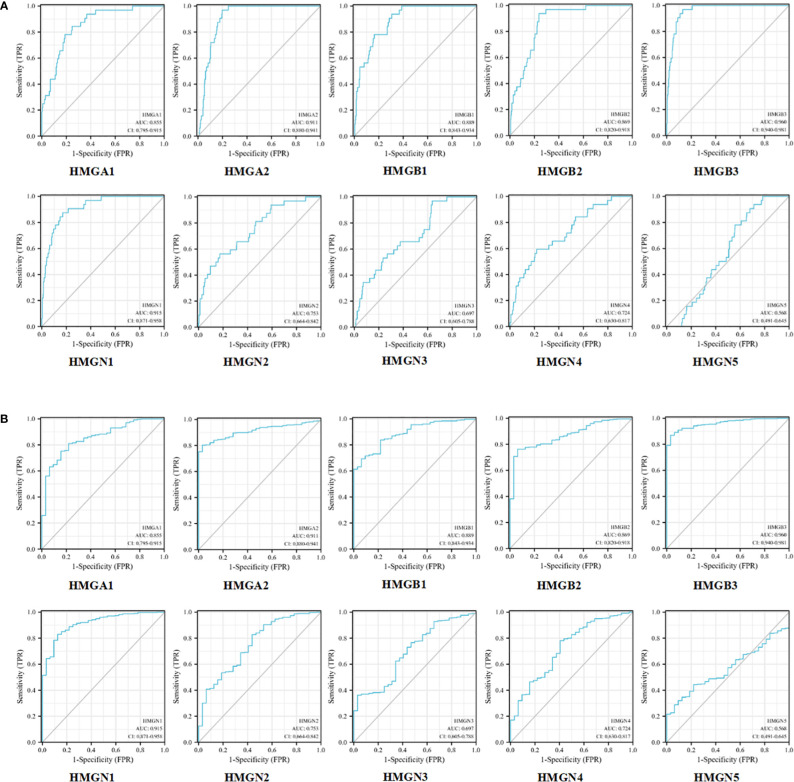
ROC curves for HMGs without **(A)** or with clinical staging **(B)** in GC.

Next, we evaluated the efficacy of HMGs as biomarkers for GC. As shown in **Figure 6A**, the mRNA expression of HMGA1 could discriminate GC from normal samples with an AUC value of 0.855 obtained from the ROC curve analysis. With a cutoff of 8.448, the sensitivity and specificity were 0.844 and 0.752, respectively. HMGA2, HMGB3, and HMGN1 showed higher accuracy in distinguishing GC from normal tissues. ROC curve analysis revealed that the AUC for HMGA2 as a diagnostic marker was 0.911 (sensitivity, 0.969; specificity, 0.803); the AUC for HMGB3 was 0.960 (sensitivity, 0.969; specificity, 0.869), and the AUC for HMGN1 was 0.915 (sensitivity, 0.875; specificity, 0.829). HMGB1 and HMGB2 showed similar values, with AUCs of 0.889 (sensitivity, 0.938; specificity, 0.693) and 0.869 (sensitivity, 0.938; specificity, 0.763), respectively. HMGN2 and HMGN4 showed some level of accuracy in distinguishing GC from normal tissues, with similar AUC values, which were 0.753 with a cutoff of 6.539 (sensitivity and specificity were 0.562 and 0.827, respectively) and 0.724 with a cutoff of 5.763 (sensitivity and specificity were 0.594 and 0.781, respectively). However, the predictive value of HMGN3 and HMGN5 in distinguishing GC from normal tissue was relatively low. ROC curve analysis showed that the AUC for HMGN3 was 0.697 (sensitivity, 0.969; specificity, 0.363) and that for HMGN5 was 0.568 (sensitivity, 0.906; specificity, 0.320). We also performed a ROC curve analysis for the predictive utility of HMGs by the clinical stage, and interestingly, consistent observations were made (**Figure 6B**). Therefore, the mRNA levels of HMGA1, HMGA2, HMGB1, HMGB2, HMGB3, HMGN1, HMGN2, and HMGN4 may serve as biomarkers to distinguish GC from normal samples. HMGA1, HMGA2, HMGB1, HMGB2, HMGB3, HMGN1, HMGN2, and HMGN4 were the potential diagnostic biomarkers for GC.

### Mutation landscape and its correlation with HMGs and PPI analysis

**Figure 7:**
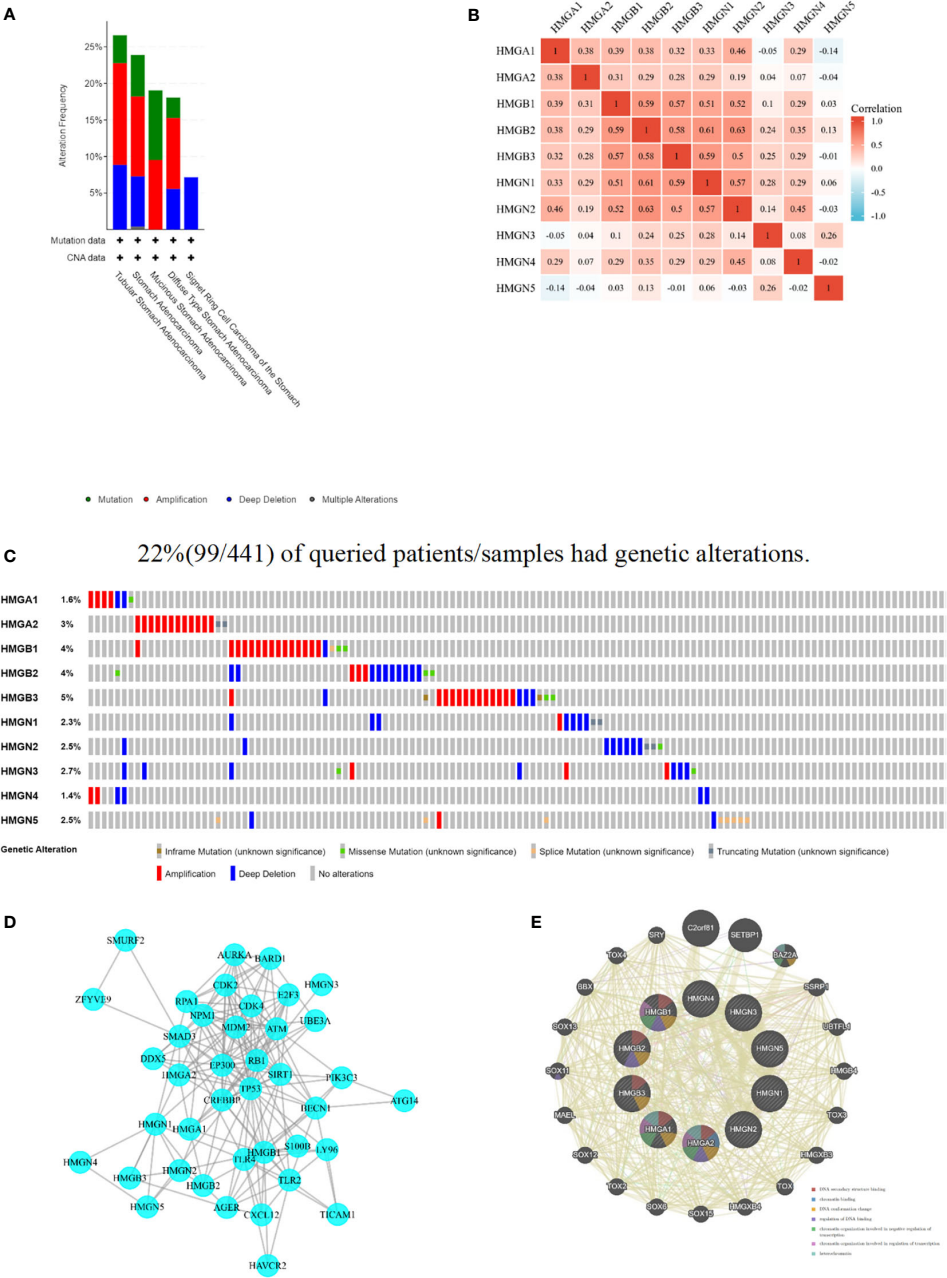
Mutation and correlation analysis of HMGs in GC. **(A)** Mutation frequency of HMGs; **(B)** correlation between every two HMGs; **(C)** Mutation specifics of every HMGs in each sample; **(D, E)** Protein-protein interaction network of different expressed HMGs.

The cBioPortal database was accessed to assess the mutational profile of HMGs. Genetic alterations occurred in 22% (99/441) of patients, and the most common mutation in the HMG subtype was gene amplification (**Figure 7A**). Specifically, except for missense mutations, splicing mutations, and deep deletions, HMGB1 was almost always carrying an amplification mutation (**Figure 7C**). Mutations in the genes of HMGA1, HMGB2, and HMGN3 included amplifications, missense, and deep deletions. HMGA2 carried both amplification and truncation mutations. HMGB3 showed amplification, missense, deep deletions, and non-frameshift mutations. Mutations in the gene for HMGN1 included deep deletions, amplifications, and truncating mutations. HMGN2 showed deep deletions, truncations, and missense mutations. HMGN4 only showed amplification mutations and deep deletions. HMGN5 not only showed amplification mutations and deep deletions but also exhibited splicing mutations. Next, we evaluated the association between HMG members by Spearman correlation analysis. As shown in **Figure 7B**, a significant positive correlation between HMGA1 and HMGA2, HMGB1, HMGB2, HMGB3, HMGN1, HMGN2, HMGN4; HMGA2 and HMGB1, HMGB3, HMGN1, HMGN2; HMGB1 and HMGB2, HMGB3, HMGN1, HMGN2, HMGN4; HMGB2 and HMGB3, HMGN1, HMGN2, HMGN3, HMGN4; HMGB3 and HMGN1, HMGN2, HMGN3, HMGN4; HMGN1 and HMGN2, HMGN3, HMGN4; HMGN2 and HMGN4, and HMGN3 and HMGN5 was found.

A PPI network was constructed using STRING to assess the potential interactors of differentially expressed HMG genes. Two nodes (40) and edges (184) were found in the PPI network (**Figure 7D**). These differentially expressed HMGs were likely to cooperate with LY96 to mediate innate immune responses against bacterial lipoproteins and other microbial cell wall components. GeneMANIA outcomes also showed that the functions of differentially expressed HMG and its related molecules (e.g., C2orf81, SETBP1, BAZ2A, SSRP1, UBTFL1, HMGB4, TOX3, HMGXB3, TOX, and HMGXB4) were mainly associated with DNA secondary structure, chromatin binding, DNA conformation changes, regulation of DNA binding, and regulation of chromatin organization involved in transcription (**Figure 7E**).

### Significant genes associated with HMGs

**Figure 8:**
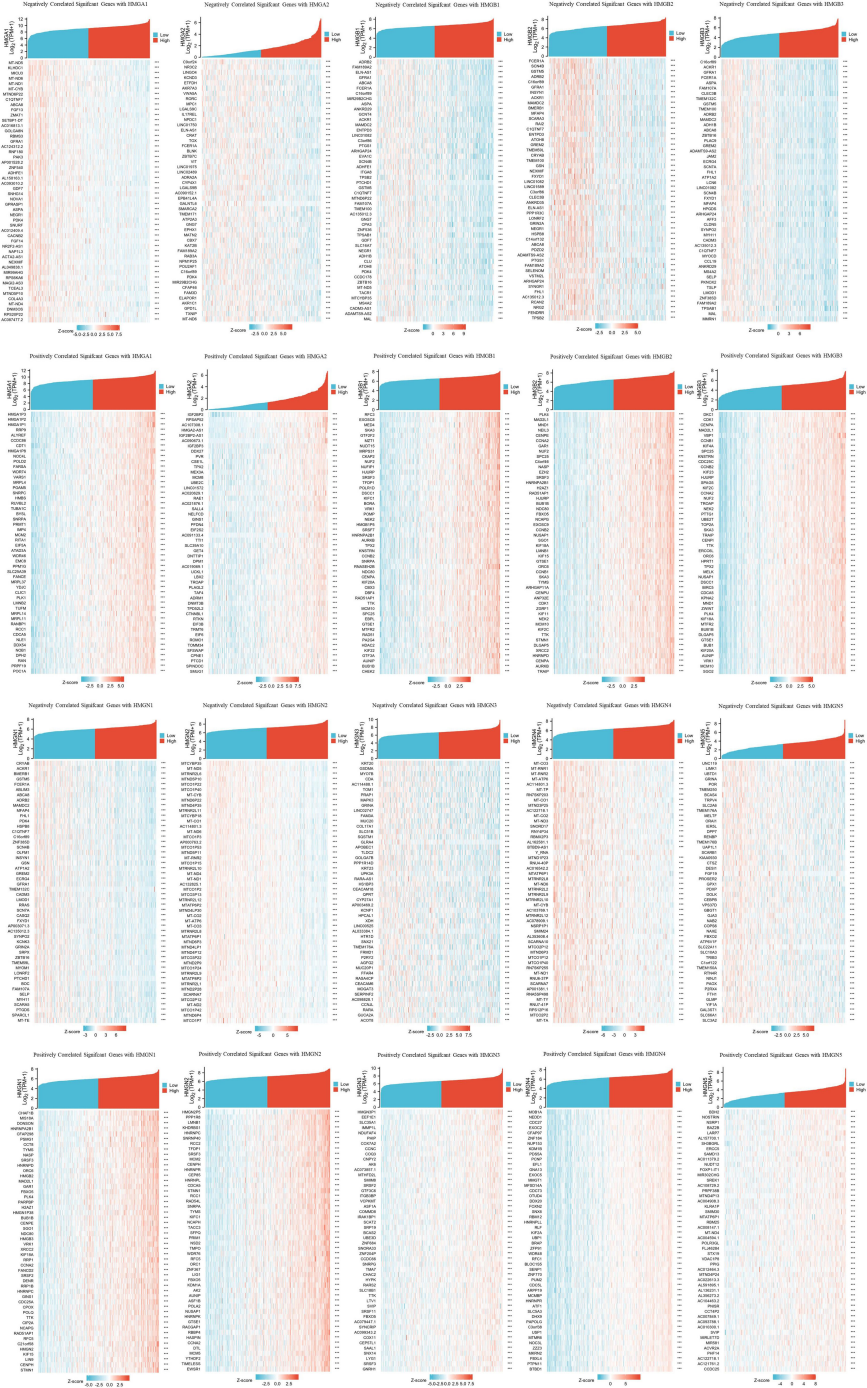
Heatmap plot of top 50 associated genes to HMGs (negatively and positively).

TCGA database was utilized to investigate important genes associated with HMG members. The top 50 genes showing the most significant association are displayed in the heatmap (**Figure 8**). The genes most negatively associated with HMGA1 included MT-ND5, KLHDC1, and MICU3, while genes most positively associated with HMGA1 were HMGA1P3, HMGA1P2 and HMGA1P1. the genes most negatively associated with HMGA2 included C9orf24, NR3C2, and LINGO4, while the genes most positively associated with HMGA2 were IGF2BP2, RPSAP52, and AC107308.1. The genes most negatively associated with HMGB1 included ADRB2, FAM189A2, and ELN-AS1, while the ones positively associated with HMGB1 were RFC3, EXOSC8, and MED4. The genes most negatively associated with HMGB2 included FCER1A, SCN4B, and GSTM5, while the ones most positively associated with HMGB2 were PLK4, MAD2L1, and MND1. The genes most negatively associated with HMGB3 included C16orf89, ACKR1, and GFRA1, while the ones most positively associated with HMGB3 were DKC1, CDK1, and CENPA. The genes most negatively associated with HMGN1 included CRYAB, ACKR1, and BMERB1, while the ones most positively associated with HMGN1 were CHAF1B, MIS18A, and DONSON. The genes most negatively associated with HMGN2 included MTCYBP35, MT-ND5, and MTRNR2L6, while the ones most positively associated with HMGN2 were HMGN2P5, PPP1R8, and LMNB1. The genes most negatively associated with HMGN3 included KRT20, GSDMA, and MYO7B, while the genes most positively associated with HMGN3 were HMGN3P1, EEF1E1, and SLC35A1. The genes most negatively associated with HMGN4 included MT-CO3, MT-RNR1, and MT-RNR2, and the genes most positively associated with HMGN4 were MOB1A, NEDD1, and CDC27. The genes most negatively associated with HMGN5 included UNC119, LIMK1, and UBTD1, while the ones most positively associated with HMGN5 were BDH2, NOSTRIN, and NSRP1.

### Correlation between immune infiltrates and HMGs

**Figure 9:**
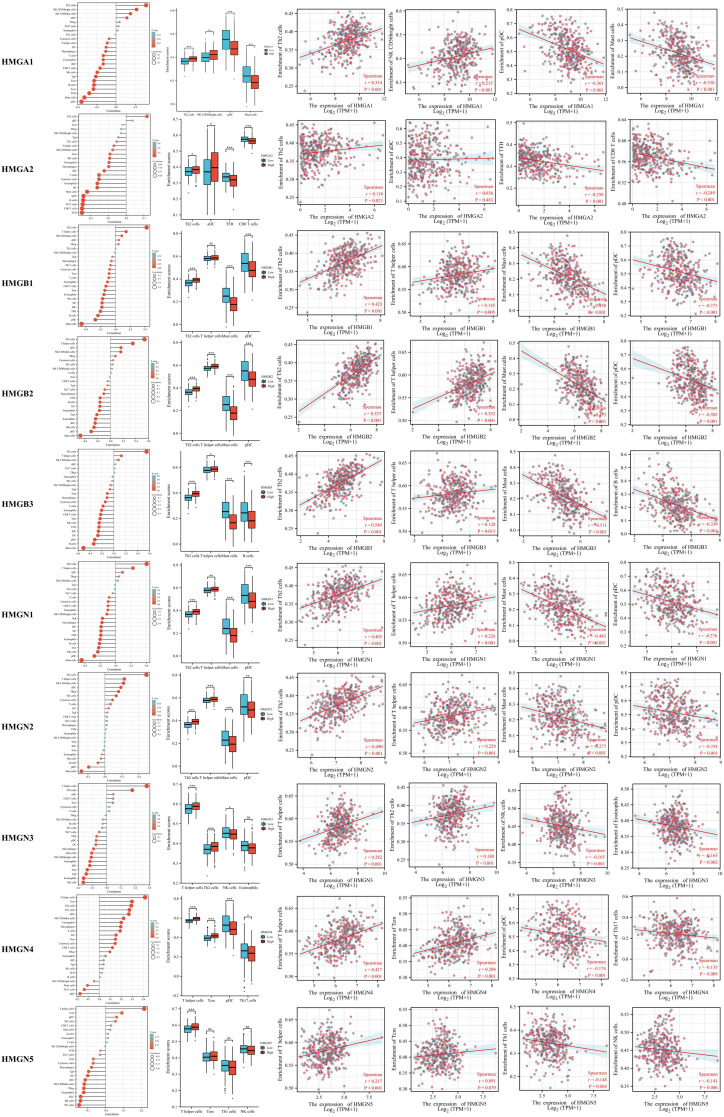
Relationship between Infiltration levels of immune cells and HMGs. Subgroup comparison plots demonstrated the first two cells that showed positive and negative correlations with HMGs. Correlation scatter plots illustrated the strength of the association between the four cells and HMGs in the subgroup comparison plots (Spearman correlation analysis). (*p< 0.05; **p<0.01; ***p<0.001).

Tumor development is closely related to immunity, and tumor-infiltrating lymphocytes perform a critical function in tumor progression, affecting the treatment and prognosis of patients with GC. Therefore, we investigated whether HMGs were associated with the level of immune infiltration in GC (**Figure 9**). The results showed that the mRNA expression of HMGA1 correlated positively with Th2 cells and NK CD56bright cells, while negatively with pDCs and mast cells. The mRNA expression of HMGA2 correlated positively with Th2 cells, while negatively with TFH and CD8 T cells. The levels of immune infiltration were similar for HMGB1, HMGB2, HMGN1, and HMGN2, and their mRNA expression correlated positively with Th2 cells and T helper cells, while negatively with mast cells and pDCs. The mRNA expression of HMGB3 correlated positively with Th2 cells and T helper cells, while negatively with mast cells and B cells. The mRNA expression of HMGN3 correlated positively with T helper cells and Th2 cells, and negatively with NK cells and eosinophils. The mRNA expression of HMGN4 correlated positively with T helper cells and Tcm, and negatively with pDCs and T17 cells. The mRNA expression of HMGN5 correlated positively with T helper cells and Tcm, while significantly negatively with Th1 cells and NK cells. Taken together, HMGs may be intimately associated with immune infiltration in GC.

### Relationship between immune checkpoints and HMGs in GC

**Figure 10:**
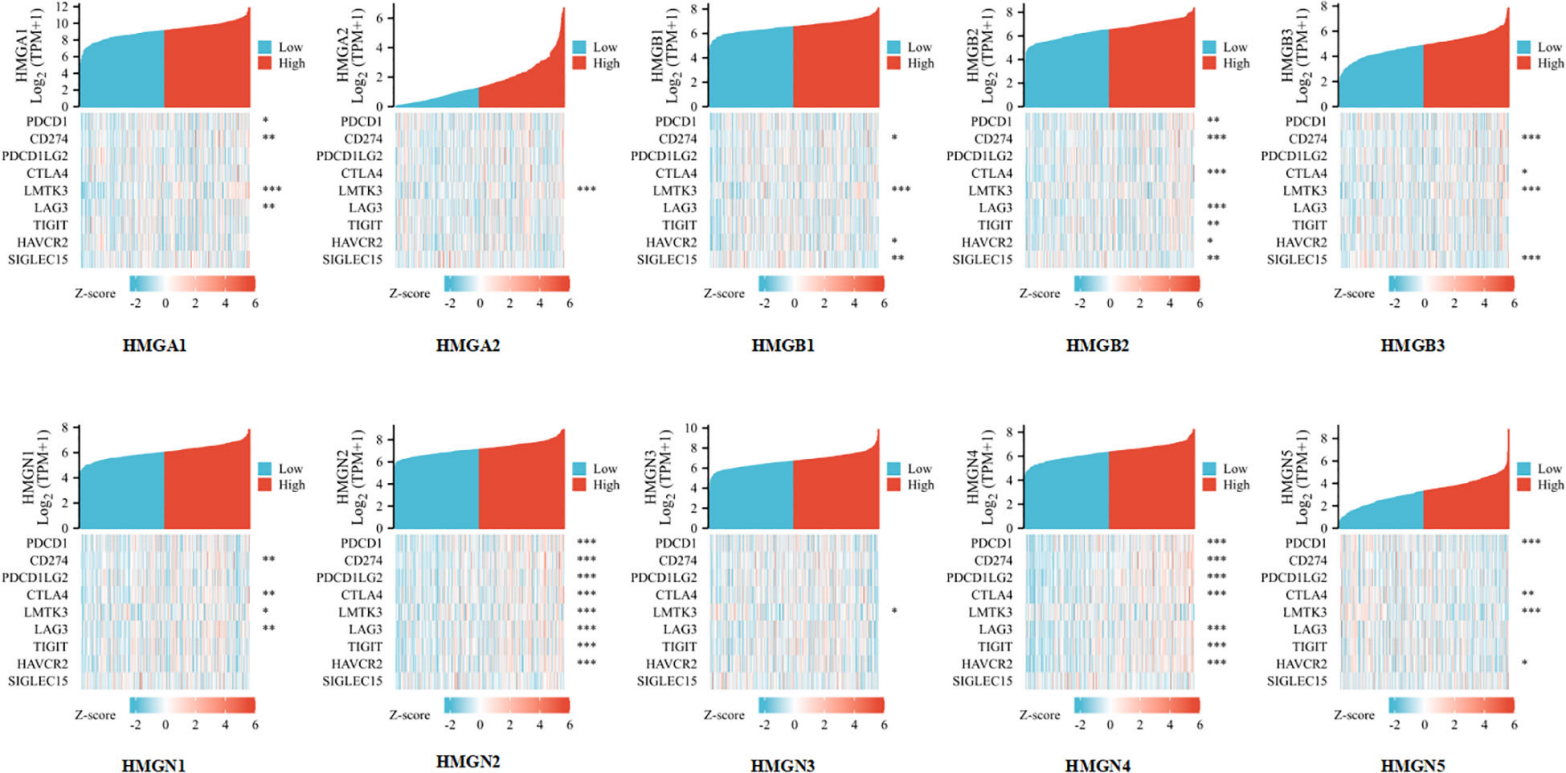
Relevance analysis of immune checkpoint-related genes and HMGs in GC (*p< 0.05; **p<0.01; ***p<0.001).

Considering that HMGs are potential oncogenes in GC, we assessed the connection of HMGs with PDCD1, CD274, PDCD1LG2, CTLA4, LMTK3, LAG3, TIGIT, HAVCR2, and SIGLEC15 (**Figure 10**). Consequently, we discovered that the level of HMGA1 expression correlated positively with PDCD1 (PD-1), CD274 (PD-L1), LMTK3, and LAG3 and the level of HMGA2 expression correlated positively with LMTK3 in GC. The level of HMGB1 expression showed a positive correlation with CD274 (PD-L1), LMTK3, HAVCR2, and SIGLEC15. The level of HMGB2 expression had a highly positive significant association with PDCD1 (PD-1), CD274 (PD-L1), CTLA4, LAG3, TIGIT, HAVCR2, and SIGLEC15 in GC. The level of HMGB3 expression had a strong positive correlation with CD274 (PD-L1), CTLA4, LMTK3, and SIGLEC15. The expression of HMGN1 correlated positively with CD274 (PD-L1), CTLA4, LMTK3, and LAG3 in GC. The expression of HMGN2 showed a particularly prominent positive relationship with PDCD1 (PD-1), CD274 (PD-L1), PDCD1LG2 (PD-L2), CTLA4, LMTK3, LAG3, TIGIT, and HAVCR2 in GC. The expression of HMGN3 showed a positive association with LMTK3 in GC. The expression of HMGN4 showed a significant positive association with PDCD1 (PD-1), CD274 (PD-L1), PDCD1LG2 (PD-L2), CTLA4, LAG3, TIGIT, and HAVCR2 in GC. The expression of HMGN5 correlated positively with PDCD1 (PD-1), CTLA4, LMTK3, and HAVCR2 in GC. These findings suggested that tumor immune escape and antitumor immunity probably participated in the oncogenic processes of GC mediated by HMGs.

### Functional analysis for HMGs

**Figure 11:**
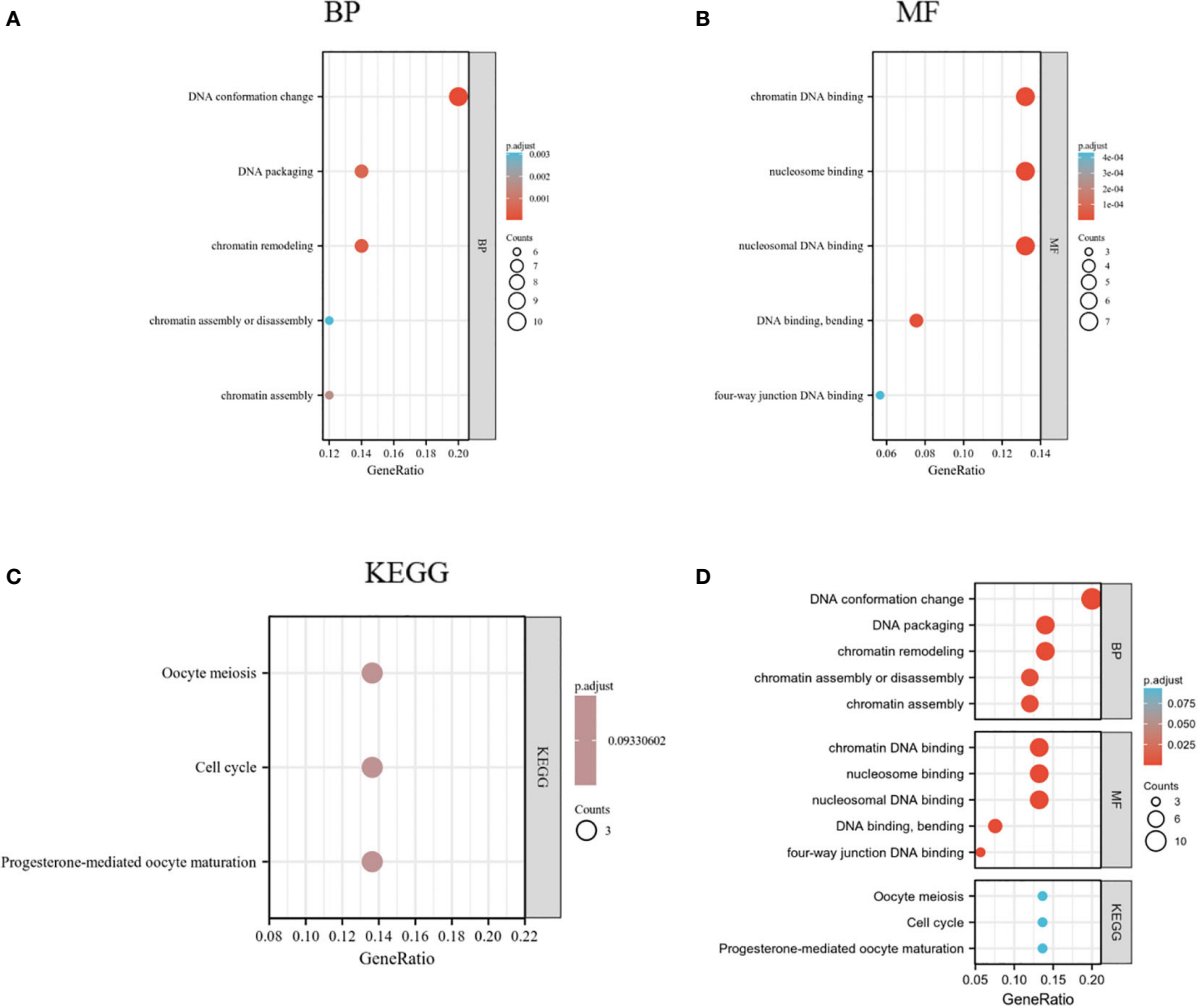
GO and KEGG enrichment analysis of HMGs. **(A)** Biological process (BP); **(B)** Molecular function (MF); **(C)** Kyoto Encyclopedia of Genes and Genomes (KEGG); **(D)** Summary plot of enrichment analysis.

HMGs and the relevant genes mentioned above (70 in total) were analyzed in the DAVID database for GO and KEGG enrichment. The top five enriched terms are shown in **Figures 11A, B**. Remarkably, the BPs were related to DNA conformation change and packaging and chromatin assembly (GO: 0071103, 0006338, 0006323, 0031497,0006333); the four MFs were associated with DNA binding (GO: 0031492, 0031490, 0008301, 0000400). These suggested that HMGs were strongly related to the cell cycle. KEGG analysis showed that the cell cycle was among the top 5 enriched pathways (**Figure 11C**). Cell cycle proteins may participate in cell growth, proliferation and differentiation, gene transcription, and DNA processes through the action of HMGs (**Supplementary Figure 1**).

## Discussion

We assayed the mRNA expression of HMGs in GC cell lines by qRT-PCR and utilizing diverse public databases, we present, herein, the first comprehensive systematic analysis of HMGs in GC. HMGA1, HMGA2, HMGB1, HMGB2, HMGB3, HMGN1, HMGN2, and HMGN4 showed high expression in GC tissues relative to normal gastric tissues. GC patients with high expression of HMGA2 and HMGN5 had shorter OS than those with low expression, suggesting the utility of HMGA2 and HMGN5 as potential prognostic prediction markers in GC. GC patients with high HMGA2 expression had shorter RFS than those with low HMGA2 expression, suggesting that HMGA2 may be a potential target for GC therapy. GO and KEGG enrichment analyses revealed that HMGs played a key function in cell cycle signaling pathways.

HMGA1 expression is of great value as a biomarker of chemotherapeutic responses in GC (60). Previously, HMGA1 expression was analyzed by immunohistochemistry in a hospital series (n = 323) comprising single hospital gastric adenocarcinoma cases (stages I to IV) with clinicopathologic and therapeutic datasets. No significant relevance of HMGA1 expression as a prognostic biomarker was noted in this collection. However, a significantly better OS was observed in cases with high HMGA1 levels following chemotherapy compared to untreated cases, implying that these patients could benefit more from treatment compared to those with low HMGA1 expression. This is in good agreement with our prediction. We collected the information from 371 patients and there was a remarkable survival difference between the high- and low-expression groups (p < 0.01). HMGA1 was highly expressed in GC cell lines, suggesting that it may promote GC. ROC curve analysis showed HMGA1’s predictive accuracy extent, and its AUC value was 0.855 with a cutoff of 8.448; the sensitivity and specificity were 0.844 and 0.752, respectively.

The HMGA2 protein might be a valuable prognostic marker for predicting tumor recurrence (61). In a study including 110 patients with primary GC, 29 adenoma samples, and 30 non-cancerous gastric tissues, HMGA2 protein levels were found to be significantly high in GC samples, and the expression correlated significantly with lymphatic invasion, peripheral nerve invasion, and TNM stage. The results of qRT-PCR revealed that HMGA2 was highly expressed in GC cell lines. HMGA2 expression was remarkably linked to shorter RFS. The above results suggested that increased HMGA2 expression may underlie carcinogenesis in GC, and was correlated with tumor cell aggressiveness and adverse prognosis of these patients. Consistently, we demonstrated that HMGA2 led to high relapse rates and may be a potential prognostic prediction marker as well as a therapeutic target in GC. However, unfortunately, HMGA2 expression was not found to be significantly associated with the tumor stage (P = 0.59). Since the data for the analysis were obtained from the GEPIA database, the findings may be attributed to the small sample size as well as the suboptimal selection of the samples or their distribution. The biological mechanisms underlying the expression of HMGA2 and its role within the GC remain largely unclear.

Previous studies show that HMGB1 is oncogenic in GC and GC patients with high HMGB1 expression exhibit a poor prognosis (27, 28). Our results also indicated that HMGB1 was highly expressed in GC cell lines, suggesting that HMGB1 might promote GC progress. We performed Kaplan-Meier Plotter analysis using the information collected from 371 patients, and OS was found to be higher in the high HMGB1 expression group than in the low HMGB1 expression group (p < 0.05), whereas HMGB1 expression was not significantly associated with RFS. This was in contrast with previous findings, and the OS was instead higher in patients with high HMGB1 expression. Jing Zhang et al. reported that knocking down HMGB1 inhibited the growth and invasion of GC cells *via* the NF-κB pathway both *in vitro* and *in vivo*, indicating that HMGB1 may serve as a potential therapeutic target for gastric adenocarcinoma (GAC) (29). Guoquan Huang et al. showed the inhibitory effect of the SEMA3B-AS1/HMGB1/FBXW7 axis in the peritoneal metastasis (PM) of GC through the modulation of biglycan (BGN) protein ubiquitination (62).

Increasing evidence suggests that HMGB2 is involved in various malignancies like prostate cancer, cervical cancer, lung cancer, melanoma, pancreatic ductal adenocarcinoma (PDAC), and GC. Pengnan Zhang et al. suggest that HMGB2 may promote cell proliferation by activating the AKT signaling pathway, thus making it a promising candidate in the search for new biomarkers and therapeutic targets in cervical cancer (63). Shugo Suzuki et al. suggest that HMGB2 expression may be a good screening tool for identifying the potential of prostate carcinogens (64). In GC, Guangfei Cui et al. found that silencing HMGB2 expression significantly decreased the proliferation and invasion of GC cells and reduced the rate of glycolysis, indicating that HMGB2 may be a novel biomarker and potential therapeutic target in GC treatment (65).

Collectively, the up-regulated expression of HMGB3 can cause the development of diverse tumors. The aberrant expression of HMGB3 suppresses the bending of the DNA and prevents the binding of DNA and transcription factors. HMGB3 can promote the production of reactive oxygen species (ROS) *via* specific Toll-like receptors (TLRs) on membranes (66), and can regulate cell cycle-induced tumorigenesis, thus promoting cancer cell proliferation and invasion by regulating signaling pathways including Wnt/β-catenin (67), MAPK (68), and Akt (69), enhancing cancer stem cell gene activity and promoting the malignant proliferation of cancer cells. HMGB3 expression is regulated by various miRNAs and long non-coding RNAs (70–72). In gastric adenocarcinoma cells, down-regulation of HMGB3 expression can dramatically suppress cancer cell proliferation, mostly through the induction of G0/G1 blockade in cancer cells, regulation of p53 and p21 signaling pathways, and downregulation of the ratio of anti-apoptotic factor Bcl-2/pro-apoptotic factor Bax (73). Moreover, the downregulation of HMGB3 expression results in the inhibition of invasion and migration of GC cells through the suppression of the activation of MMP2 and MMP9 (74). These results suggest that HMGB3 is an emerging tumor diagnostic and prognostic marker protein (75).

HMGN1 functions as a nucleosome-binding protein that regulates chromatin structure, histone modifications, and gene expression. Jae-Hwan Lim et al. found that HMGN1 could enhance acetylation of lysine 14 in histone H3, thus playing an important role in chromatin regulation (76). Yehudit Birger et al. revealed that HMGN1 increased the repair rate of ultraviolet (UV)-induced DNA lesions in chromatin, confirming the role of HMGN1 in DNA damage repair (77). HMGN1 is an alert protein contributing to the extracellular production of LPS-induced innate and antigen-specific (78) as well as Th1-polarized adaptive immune responses (79), which play a key role in cell-mediated tumor immune responses. Owing to the ability to boost DNA repair and prioritize Th1 immune responses, HMGN1 is an ideal candidate for a vaccine adjuvant and developing antitumor therapies (80).

The abnormal expression of HMGN2 is often closely related to the occurrence and development of tumors. HMGN2 can inhibit the proliferation and cell cycle of tumor cells in breast cancer (81), oral squamous cell carcinoma (82), and osteosarcoma (83). Kimmo Porkka et al. show (84) that the nucleosome-binding domain F3 peptide of HMGN2 is a promising potential tumor therapeutic target, suggesting unique prospects for drug-targeting applications because it can be absorbed by cells and carry payloads to the nucleus. The C terminus of HMGN2 is similar to the structure of tumor invasion inhibitory factor 2 (IIF2). The inhibition rate of tumor metastasis in mice with lung cancer injected with IIF2 is 50–60%, further suggesting that HMGN2 can suppress tumor metastasis (85). Its N terminus can selectively bind to tumor cells (86). These results suggest that the N-terminal peptide of HMGN2 can carry cytotoxic agents into tumor cells, while the C-terminal peptide can be used to develop drugs to inhibit tumor metastasis. The unique structure and targeting ability of HMGN2 is expected to provide a new direction for the development of anticancer drugs.

HMGN4 is widely and differentially expressed in various human tissues, with higher HMGN4 mRNA levels in the thyroid gland, thymus, and lymph nodes and lower expression in the liver, pancreas, testis, and embryo. HMGN4 upregulation in mouse and human cells and the thyroid gland of transgenic mice alters cellular transcriptional profiles, downregulates the expression of the tumor suppressors, including Atm, Atrx, and Brca2, and elevates the level of the DNA damage-marker, γH2AX. Jamie Kugler et al. identified HMGN4 as a new epigenetic factor that enhanced thyroid carcinogenesis and raised its potential as a diagnostic marker or target for treatment for certain thyroid cancers (53). HMGN4 plays a critical function in STAT3-mediated carcinogenesis in triple-negative breast cancer (TNBC) and it may well be a potential new focus for anti-TNBC therapy (54).

Although the levels of HMGN3 and HMGN5 expression in GC and normal tissues were statistically significant, they were comparable. At present, little is known about their exact functions in GC. Only HMGN5 has been reported to play an oncogenic role in GC, whereby it promotes GC cell growth (87). HMGN5 was highly expressed in GC cell lines, contrary to the predictions using TCGA cohort. However, it was consistent with the results of the above experimental study. This indicated that some PTMs of HMGN5 mRNA necessarily occurred at the stage of mRNA translation to protein, correspondingly resulting in lower protein levels. HMGN5 is scantily studied and genetic and clinical evidence is needed to assess its value.

Some limitations of this study warrant further consideration. First, the study lacked experimental validation and molecular experiments. Second, GC showed strong heterogeneity (88), whereas mRNA expression in TCGA database was average across all cell types of the tumor. Further elucidation of the role of HMGs in GC by single-cell sequencing analyses is required.

## 5 Conclusion

In summary, we systematically analyzed the differential expression, immune infiltration, and prognostic value of HMGs in GC. Except for HMGN3 and HMGN5, the levels of expression of the remaining members were significantly enhanced in GC. Our findings strongly suggested the vital role of HMGs in GC. HMGA2 and HMGB3 could be potential markers for prognostic prediction and serve as therapeutic targets for the treatment of GC patients by interrupting pathways underlying the cell cycle. Our research may provide renewed perspectives for prognostic biomarker selection among HMGs in GC and their future utilization may contribute to the determination of the optimal strategy for the treatment of these patients.

## Data availability statement

Publicly available datasets were analyzed in this study. This data can be found here: https://xenabrowser.net/datapages/, https://portal.gdc.cancer.gov/.

## Author contributions

ZW, YH, and WY: searched data, did the experiments, analyzed data and results, and wrote the manuscript. XW and HS: analyzed data and results. ZW, ML and AX: edited the manuscript. AX and ML: conception of the project. All authors contributed to the article and approved the submitted version.

## Glossary

**Table:**

ACC	Adrenocortical Carcinoma
BLCA	Bladder Urothelial Carcinom
BRCA	Breast Invasive Carcinoma
CESC	Cervical Squamous Cell Carcinoma and Endocervical Adenocarcinoma)
CHOL	Cholangio Carcinoma
COAD	Colon Adenocarcinoma
DLBC	Lymphoid Neoplasm Diffuse Large B-cell Lymphoma
ESCA	Esophageal Carcinoma
GBM	Glioblastoma Multiforme
HNSC	Head and Neck Squamous Cell Carcinoma
KICH	Kidney Chromophobe
KIRC	Kidney Renal Clear Cell Carcinoma
KIRP	Kidney Renal Papillary Cell Carcinoma
LAML	Acute Myeloid Leukemia
LGG	Brain Lower Grade Glioma
LIHC	Liver Hepatocellular Carcinoma
LUAD	Lung Adenocarcinoma
LUSC	Lung Squamous Cell Carcinoma
MESO	Mesothelioma
OV	Ovarian Serous Cystadenocarcinoma
PAAD	Pancreatic Adenocarcinoma
PCPG	Pheochromocytoma and Paraganglioma
PRAD	Prostate Adenocarcinoma
READ	Rectum Adenocarcinoma
SARC	Sarcoma
SKCM	Skin Cutaneous Melanoma
STAD	Stomach Adenocarcinoma
TGCT	Testicular Germ Cell Tumors
THCA	Thyroid Carcinoma
THYM	Thymoma
UCEC	Uterine Corpus Endometrial Carcinoma
UCS	Uterine Carcinosarcoma
UVM	Uveal Melanoma
AUC	Area Under Curve
BGN	biglycan
DAMP	damageassociated molecular pattern
GAC	gastric adenocarcinoma
GAPDH	Glyceraldehyde-3-phosphate dehydrogenase
GC	gastric cancer
GO	Gene Ontology
HMGs	High mobility group proteins
HMGB	high mobility group protein B
HMGN	high mobility group nucleosome binding protein
IIF2	invasion inhibitory factor 2
KEGG	Kyoto Encyclopedia of Genes and Genomes
OS	overall survival
PAMP	pathogen-associated molecular pattern
PDAC	pancreatic ductal adenocarcinoma
PM	peritoneal metastasis
PTMs	protein translational modifications
RFS	recurrence-free survival
ROC	receiver operating characteristic
ROS	reactive oxygen species
TLRs	the Toll-like receptors
TNBC	triple-negative breast cancer
TCGA	The Cancer Genome Atlas
GTEx	Genotype-Tissue Expression Project
