# Supplementary material for: Expression, tumor immune infiltration, and prognostic impact of HMGs in gastric cancer
Source: Front Oncol. 2022 Dec 7;12:1056917. doi: 10.3389/fonc.2022.1056917 (PMC9780705; doi:10.3389/fonc.2022.1056917)
Supplement: Supplementary file 1 [file DataSheet_1.pdf]

Genes expression difference analysis:

```
library(tidyverse)
library(ggplot2)
library(reshape2)
library(car)
library(rstatix)

set.seed(100)
data <- data.frame(x = rnorm(100, 2, 1), y = rnorm(100, 1, 1))
data2 <- melt(data)

data3 <- lapply(data, function(x) get_summary_stats(data.frame(x)))
data3

data3 <- rbind(data3[[1]], data3[[2]])
data3[1] <- c("x", "y")

## Shapiro-Wilk normality test
lapply(data, function(x) shapiro.test(x))

## Levene's Test
leveneTest(value~variable, data = data2)


wilcox.test(value~variable, data = data2)
# Wilcoxon rank sum test with continuity correction


summary(aov(value~variable, data = data2))
# Df Sum Sq Mean Sq F value   Pr(>F)


ggplot(data2, aes(x = variable, y = value, color = variable, fill = variable)) +
  geom_violin(alpha = 0.2) +
  theme_bw()

ggplot(data2, aes(x = variable, y = value, color = variable, fill = variable)) +
  geom_violin(alpha = 0.2) +
  geom_point(position = position_jitter(0.3)) +
  theme_bw()

ggplot(data2, aes(x = variable, y = value, color = variable, fill = variable)) +
  geom_boxplot(alpha = 0.2) +
  geom_point(position = position_jitter(0.3)) +
  theme_bw()
```

```
ggplot(data2, aes(x = variable, y = value, color = variable, fill = variable)) +
  geom_violin(alpha = 0.1) +
  geom_boxplot(alpha = 0.1) +
  geom_point(position = position_jitter(0.3)) +
  theme_bw()

ggplot() +
  geom_violin(data = data2, aes(x = variable, y = value, color = variable, fill =
variable), alpha = 0.1) +
  geom_errorbar(data = data3, aes(x = variable, ymin=mean-sd, ymax=mean+sd), width =
0.2)
```

The receiver operating characteristic (ROC) curves:

```
library(tidyverse)
library(pROC)
library(ggplot2)
library(reshape2)
library(rstatix)

# data <- dat
data <- read.table("~/file.txt", header = T)
data$outcome <- factor(data$outcome, levels = c("group1", "group2"))
head(data)
#   outcome        a         b        c


data2 <- gather(data, key = "x", value = "value", -outcome)
data3 <- data2 %>%
  group_by(outcome, x) %>%
  get_summary_stats(value)
data3
#  outcome x      variable     n   min    max median    q1    q3   iqr   mad  mean
sd    se    ci

roc1 <- roc(response = data$outcome, predictor = data$a)
# Call:
#   roc.default(response = data$outcome, predictor = data$a)
#
# Data: data$a in 40 controls (data$outcome group1) > 32 cases (data$outcome group2).
# Area under the curve:
roc2 <- roc(response = data$outcome, predictor = data$b)
# Call:
#   roc.default(response = data$outcome, predictor = data$b)
#
# Data: data$b in 40 controls (data$outcome group1) > 32 cases (data$outcome group2).
```

```
# Area under the curve:
roc3 <- roc(response = data$outcome, predictor = data$c)
# Call:
#   roc.default(response = data$outcome, predictor = data$c)
#
# Data: data$c in 40 controls (data$outcome group1) > 32 cases (data$outcome group2).
# Area under the curve:

ci.auc(roc1)
# 95% CI:
ci.auc(roc2)
# 95% CI:
ci.auc(roc3)
# 95% CI:


coords(roc1, x =  "best", ret="all")
#             threshold specificity sensitivity  accuracy tn tp fn fp        npv
ppv       fdr  fpr   tpr  tnr   fnr
# threshold
#            1-specificity 1-sensitivity 1-accuracy      1-npv      1-ppv precision
recall youden closest.topleft
# threshold


roc.test(roc1, roc2, reuse.auc=FALSE, method = "delong")
# DeLong's test for two correlated ROC curves
#
# data:  roc1 and roc2
# Z = , p-value =
# alternative hypothesis: true difference in AUC is not equal to 0
# sample estimates:
# AUC of roc1 AUC of roc2


plot.roc(roc1)


data1 <- data.frame(group = "a",
                  x = 1-roc1$specificities,
                  y = roc1$sensitivities)
data1 <- data1[order(data1$x, data1$y),]
tmp <- data.frame(group = "b",
                    x = 1-roc2$specificities,
                    y = roc2$sensitivities)
tmp <- tmp[order(tmp$x, tmp$y),]
data1 <- rbind(data1, tmp)
```

```
data1$group <- factor(data1$group, levels = c("a", "b"))


ggplot() +
  geom_line(data = data1, aes(x = x, y = y, colour = group)) +
  labs(x = "1-Specificity (FPR)", y = "Sensitivity (TPR)")
```

Heat map correlational analyses:

```
library(tidyverse)
library(ggplot2)
library(patchwork)
library(reshape2)


data <- read.table("~/file.txt", header = T)
head(data)

data$group <- ifelse(data$target >= median(data$target), "High", "Low")
data$group <- factor(data$group, levels = c("Low", "High"))
data<- data[order(data$target), ]
data$id <- 1:nrow(data)


### plot
p1 <- ggplot() +
  geom_bar(data = data,
           aes(x = id, y = target, color=group, fill = group),
           stat = 'identity', position = 'dodge') +
  scale_y_continuous(expand = c(0,0)) +
  theme_classic() +
  theme(axis.title.x = element_blank(), axis.text.x = element_blank(),
        axis.ticks.x = element_blank(), axis.line.x = element_blank())

data1 <- data %>%
  select(-group, -target) %>%
  remove_rownames() %>%
  column_to_rownames("id") %>%
  scale() %>% as.data.frame() %>%
  rownames_to_column("id") %>%
  melt()

p2 <-
ggplot(data = data1, aes(x = id, y = as.numeric(variable), fill = value)) +
  geom_raster() +
  scale_fill_gradientn(colors = c("#4DBBD5", "#FFFFFF", "#E64B35")) +
```

```
  scale_y_continuous(expand = c(0,0), limits = c(0.5,5.5), breaks = 1:5,
                     labels = levels(data1$variable)) +
  theme(axis.title = element_blank(), axis.text.x = element_blank(),
        axis.ticks.x = element_blank(), axis.line.x = element_blank())

p1 / p2
```

Immune infiltration analysis (lollipop chart):

```
# library(tidyverse)
library(GSVA)
library(clusterProfiler)
library(org.Hs.eg.db)
library(data.table)
library(rtracklayer)

### ssGSEA ######
## table S1 - https://doi.org/10.1016/j.immuni.2013.10.003
## pdf -> table -> read
immunity <- read.csv("~/immunity-cell-gene.csv", header = T)

idx <- !immunity$CellType %in% c("Blood vessels", "Normal mucosa", "SW480 cancer cells",
"Lymph vessels")
immunity <- immunity[idx,]
immunity <- immunity %>%
  split(., .$CellType) %>%
  lapply(., function(x)(x$ENTREZ_GENE_ID))
immunity <- lapply(immunity, unique)

## Ensembl download
anno <- import('~/Homo_sapiens.GRCh38.101.gtf')
anno <- as.data.frame(anno)
anno <- anno[!duplicated(anno$gene_id),]

anno <- merge(anno, gene_symbol, by = "gene_name")
anno <- rbind(anno, data.frame(gene_name = c(" ", " "),
                               gene_id = c("ENSG ", "ENSG "),
                               ENTREZID = c(" ", " ")))
anno <- anno[!duplicated(anno$gene_id),] ### 37417
anno <- anno[, c("gene_id", "ENTREZID")]


data <- fread("~/tpm.txt") %>%
  rename("gene_id" = "V1") %>%
  left_join(., anno, by = "gene_id") %>%
  filter(!is.na(ENTREZID)) %>%
```

```
  select(-gene_id) %>%
  column_to_rownames("ENTREZID")
data <- log2(data + 1)

immu_cell <-  as.data.frame(gsva(as.matrix(data), immunity, method = "ssgsea"))


### bbt plot
data <- read.table("~/file.txt", header = T)

data1 <- NULL
for(i in 2:25){
  cor <- cor.test(data[,i], data[,1], method = "pearson")
  data1 <- rbind(data1,
                 data.frame("group" = "a",
                            "cell" = colnames(data)[i],
                            "cor" = cor$estimate,
                            "p" = cor$p.value))
}
data1 <- data1[order(data1$cor),]
data1$cell <- factor(data1$cell, levels = data1$cell)

ggplot(data1, aes(x = cell, y = cor)) +
  geom_segment(aes(xend=cell,yend=0)) +
  geom_hline(yintercept = 0) +
  geom_point(aes(col=p, fill = p, size=abs(cor))) +
  coord_flip()
```

Immune infiltration analysis (subgroup comparison chart):

```
# library(tidyverse)
library(GSVA)
library(clusterProfiler)
library(org.Hs.eg.db)
library(data.table)
library(rtracklayer)

### ssGSEA ######
## table S1 - https://doi.org/10.1016/j.immuni.2013.10.003
## pdf -> table -> read
immunity <- read.csv("~/immunity-cell-gene.csv", header = T)

idx <- !immunity$CellType %in% c("Blood vessels", "Normal mucosa", "SW480 cancer cells",
"Lymph vessels")
immunity <- immunity[idx,]
immunity <- immunity %>%
```

```
  split(., .$CellType) %>%
  lapply(., function(x)(x$ENTREZ_GENE_ID))
immunity <- lapply(immunity, unique)

## Ensembl download
anno <- import('~/Homo_sapiens.GRCh38.101.gtf')
anno <- as.data.frame(anno)
anno <- anno[!duplicated(anno$gene_id),]

anno <- merge(anno, gene_symbol, by = "gene_name")
anno <- rbind(anno, data.frame(gene_name = c(" ", " "),
                               gene_id = c("ENSG ", "ENSG "),
                               ENTREZID = c(" ", " ")))
anno <- anno[!duplicated(anno$gene_id),] ### 37417
anno <- anno[, c("gene_id", "ENTREZID")]


data <- fread("~/tpm.txt") %>%
  rename("gene_id" = "V1") %>%
  left_join(., anno, by = "gene_id") %>%
  filter(!is.na(ENTREZID)) %>%
  select(-gene_id) %>%
  column_to_rownames("ENTREZID")
data <- log2(data + 1)

immu_cell <-  as.data.frame(gsva(as.matrix(data), immunity, method = "ssgsea"))


### group plot
data <- read.table("~/file.txt", header = T)

colnames(data) <- c("x", "y")
data$group <- ifelse(data$x >= median(data$x), "High", "Low")
data$group <- factor(data$group, levels = c("Low", "High"))

ggplot(data, aes(x = group, y = y, color = group, fill = group)) +
  geom_boxplot(alpha = 0.2) +
  geom_point(position = position_jitter(0.3)) +
  theme_bw()
```

Immune infiltration analysis (scatter chart):

```
# library(tidyverse)
library(GSVA)
library(clusterProfiler)
```

```r
library(org.Hs.eg.db)
library(data.table)
library(rtracklayer)

### ssGSEA ######
## table S1 - https://doi.org/10.1016/j.immuni.2013.10.003
## pdf -> table -> read
immunity <- read.csv("~/immunity-cell-gene.csv", header = T)

idx <- !immunity$CellType %in% c("Blood vessels", "Normal mucosa", "SW480 cancer cells",
"Lymph vessels")
immunity <- immunity[idx,]
immunity <- immunity %>%
  split(., .$CellType) %>%
  lapply(., function(x)(x$ENTREZ_GENE_ID))
immunity <- lapply(immunity, unique)

## Ensembl download
anno <- import('~/Homo_sapiens.GRCh38.101.gtf')
anno <- as.data.frame(anno)
anno <- anno[!duplicated(anno$gene_id),]

anno <- merge(anno, gene_symbol, by = "gene_name")
anno <- rbind(anno, data.frame(gene_name = c(" ", " "),
                               gene_id = c("ENSG ", "ENSG "),
                               ENTREZID = c(" ", " ")))
anno <- anno[!duplicated(anno$gene_id),] ### 37417
anno <- anno[, c("gene_id", "ENTREZID")]


data <- fread("~/tpm.txt") %>%
  rename("gene_id" = "V1") %>%
  left_join(., anno, by = "gene_id") %>%
  filter(!is.na(ENTREZID)) %>%
  select(-gene_id) %>%
  column_to_rownames("ENTREZID")
data <- log2(data + 1)

immu_cell <-  as.data.frame(gsva(as.matrix(data), immunity, method = "ssgsea"))


### cor plot
data <- read.table("~/file.txt", header = T)

colnames(data) <- c("x", "y")

ggplot(data, aes(x = x, y = y)) +
```

```
geom_point() +
geom_smooth(formula = y ~ x, method = "lm") +
theme_bw()
```
