# Supplementary figures and images for: Expression, tumor immune infiltration, and prognostic impact of HMGs in gastric cancer
Source: Front Oncol. 2022 Dec 7;12:1056917. doi: 10.3389/fonc.2022.1056917 (PMC9780705; doi:10.3389/fonc.2022.1056917)

## Supplementary figure legends

Supplementary Figure 1. Cell cycle correlated with HMGs

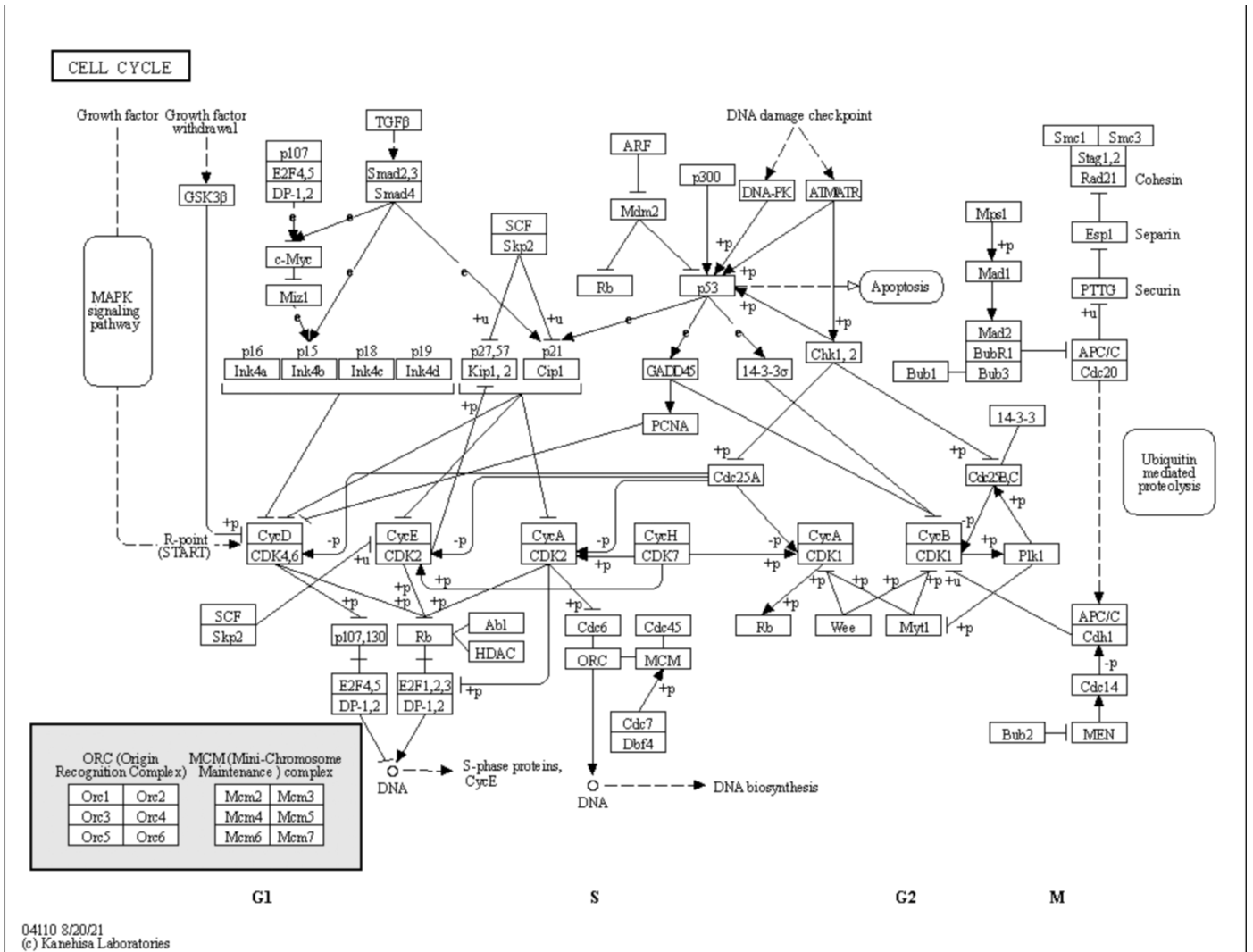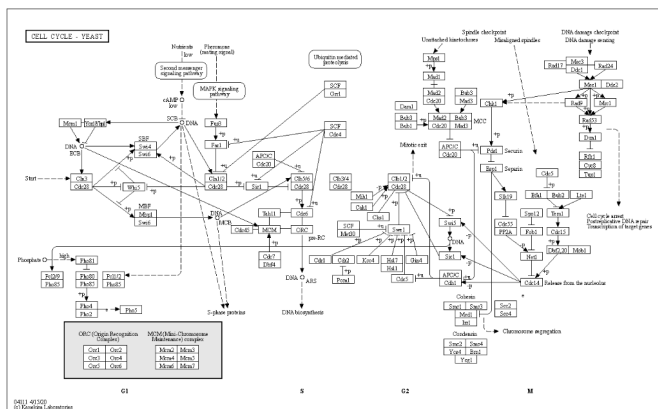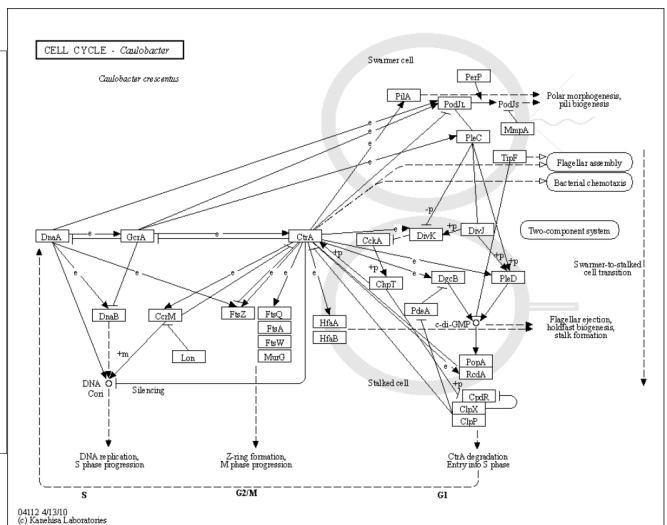

Supplement: Supplementary file 2 [file DataSheet_2.pdf]
